# Studying the Outcomes in Patients with Tricuspid Regurgitation Treated with Valve Repair or Valve Replacement: Interpreting the Survival Pattern on The Long Term by Application of Artificial Intelligence Methods

**DOI:** 10.31083/j.rcm2506223

**Published:** 2024-06-20

**Authors:** Andrea Messori, Sabrina Trippoli, Maria Rita Romeo, Valeria Fadda, Melania Rivano, Lorenzo Di Spazio

**Affiliations:** ^1^HTA Unit, Regione Toscana, Regional Health Service, 50139 Firenze, Italy; ^2^Ospedale del Cuore, Fondazione Monasterio, Regional Health Service, 54100 Massa, Italy; ^3^ESTAR Toscana, Regional Health Service, 50135 Firenze, Italy; ^4^Hospital Pharmacy Department, R. Binaghi, 09126 Cagliari, Italy; ^5^Scientific Direction of Italian Society of Clinical Pharmacy and Therapeutics (SIFACT), 10123 Torino, Italy; ^6^Hospital Pharmacy Department, S. Chiara Trento Hospital, Azienda Provinciale per i Servizi Sanitari (APSS), 38122 Trento, Italy

**Keywords:** meta-analysis, kaplan-meier curves, ipdfromkm method, artificial intelligence, reconstructed individual patient data

## Abstract

**Background:**

The reconstruction of individual patient data from published Kaplan-Meier survival curves is a new technique (often denoted as the IPDfromKM method) for studying efficacy in cases where multiple trials are available, and the endpoint is long-term mortality. In patients with tricuspid regurgitation, both valve repair and valve replacement have been proposed to improve prognosis; 6 controlled clinical trials (CTs) have been conducted to compare the two therapeutic options mentioned above. The objective of our analysis was to study these six trials through the application of the IPDfromKM method.

**Methods:**

In the present report, we applied the IPDfromKM method to carry out a pooled analysis of these 6 CTs to investigate the effectiveness of valve repair vs valve replacement and to assess the between-study heterogeneity from this clinical material. After reconstructing individual patient data from these 6 trials, patients treated with valve repair were pooled together and their Kaplan-Meier curve was generated. Likewise, patients treated with valve replacement were pooled together and their Kaplan-Meier curve was generated. Finally, these two curves were compared by standard survival statistics. The hazard ratio (HR) was determined; death from any cause was the endpoint.

**Results:**

These 6 CTs included a total of 552 patients; in each of these CTs, the patient group treated with valve repair was compared with another group treated with valve replacement. Our statistical results showed a significantly better survival for valve repair compared with valve replacement (HR, 0.6098; 95% confidence intervals (CI), 0.445 to 0.835; *p* = 0.002). Heterogeneity was found to be significant in the 6 patient arms undergoing replacement, but not in those undergoing valve repair. In valve replacement, the classification of patients in class III or IV of New York Heart Association (NYHA) was the main negative prognostic factor.

**Conclusions:**

Our analysis confirmed the methodological advantages of the IPDfromKM method in the indirect comparative analysis of multiple trials. These advantages include appropriate analysis of censored patients, original assessment of heterogeneity, and graphical presentation of the results, wherein individual patients retain an important role.

## 1. Introduction

In patients diagnosed with tricuspid regurgitation, interventions such as tricuspid valve repair (VREPA) and valve replacement (VREPLA) have been proposed to improve long-term prognosis [1, 2]. Several controlled trials (CTs) have compared the outcomes of VREPA and VREPLA over extended follow-up periods [3, 4, 5, 6, 7, 8]. Notably, the design of these trials was primarily observational and none of them used a randomised design.

Over the past two years, there has been a notable increase in the use of a novel method known as IPDfromKM (reconstruct individual patient data from published Kaplan-Meier survival curves) [9] to aggregate survival results from various clinical trials. Initially developed for use in oncology [10], this method has gained significant traction in cardiology [11]. The IPDfromKM method is unique in that it uses artificial intelligence techniques to analyse Kaplan-Meier curves [10, 11]. This approach allows individual patient data to be reconstructed with remarkable accuracy, as has been demonstrated in numerous publications.

In this current report, we used the IPDfromKM method to perform a pooled analysis of the trials that compared VREPA and VREPLA [3, 4, 5, 6, 7, 8]. Our aim was to assess the between-study heterogeneity arising from the analysis of these trials.

## 2. Materials and Methods

### 2.1 Literature Search

Our Pubmed search, based on the keywords “tricuspid AND regurgitation AND (repair OR replacement)” with filters on “Clinical trials” and “last 10 years”, was run on 25 November 2023. The selection of pertinent articles was managed through the PRISMA algorithm [12]. Inclusion criteria focused on any clinical trial reporting original effectiveness data for both valve repair and valve replacement in patients with tricuspid valve regurgitation. Survival had to be the endpoint of the clinical trial. The only endpoint considered in our analysis was death from any cause. The availability of a Kaplan-Meier curve with the survival results was also a mandatory criterion. The minimum follow-up was 24 months.

### 2.2 Survival Analysis

Each of the included trials was subjected to a standard IPDfromKM analysis [9, 10, 11, 13, 14]. In each of these trials, our analysis examined the two treatments (VREPA and VREPLA) and compared them with one another based on the endpoint mentioned above. All analyses were performed on reconstructed individual patient data. The statistical comparisons were performed according to the hazard ratio (HR); 95% confidence intervals (CI) were determined where appropriate. In running the IPDfromKM analysis, all patient arms treated with VREPA were pooled together to generate a single survival curve. Likewise, all patient arms treated with VREPLA were pooled. Finally, the two pooled curves for VREPA and VREPLA, respectively, were compared based on standard survival statistics (i.e., Cox model, HR with 95% CI, etc.). For this purpose, four packages (“survival”, “survminer”, “survRM2”, and “readxl”) of the R-platform [15] were used. Censored patients were managed through standard methods. In two separate analyses, heterogeneity was assessed through the typical approach required by the IPDfromKM method [10, 11, 13, 14]. Hence, for each trial the survival curves of reconstructed patients were plotted in a single graph that included all patient arms employing the same treatment (i.e., VREPA or VREPLA). In these graphs, between-trial differences were assessed both visually and on the basis of standard statistical indexes (e.g., likelihood ratio test).

## 3. Results

### 3.1 Literature Search

**Fig. 1.**
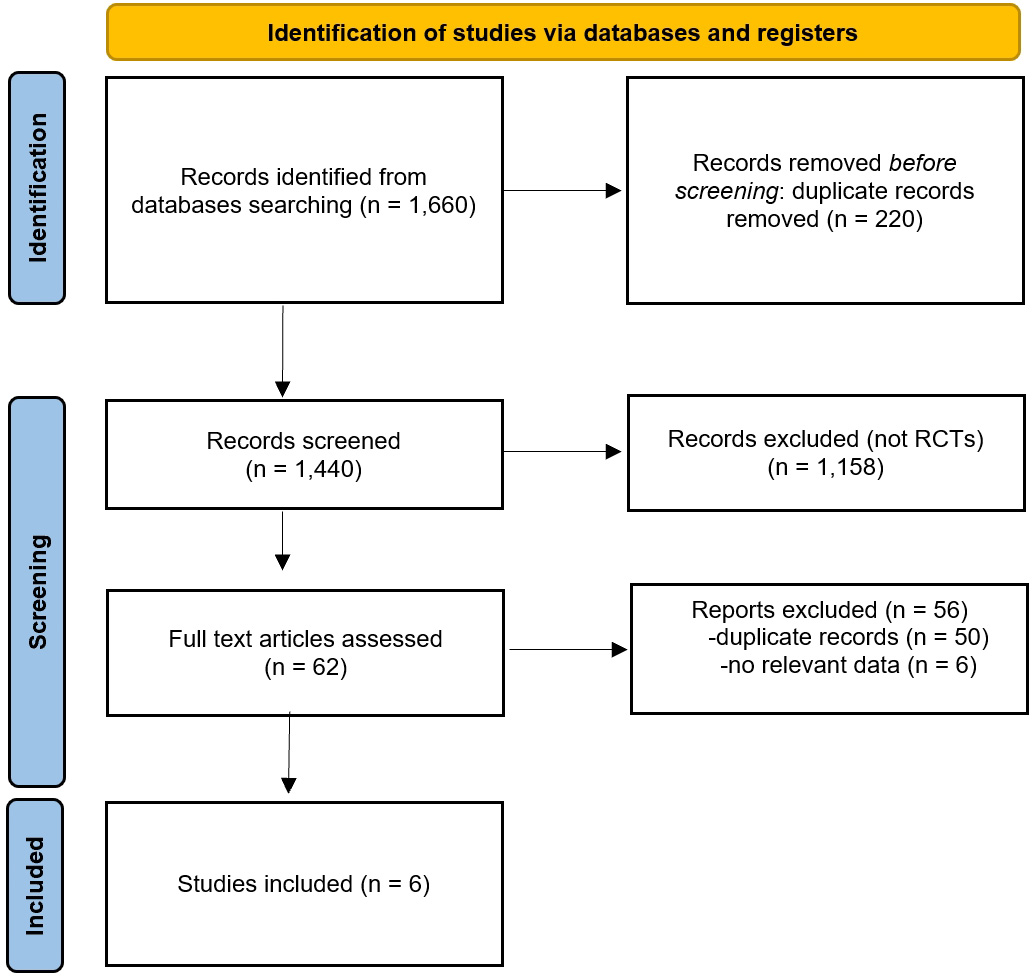
**Preferred Reporting Items for Systematic Reviews and Meta-analyses (PRISMA) flow diagram.** The keywords employed in our PubMed search were the following: atrial AND fibrillation AND (radiofrequency OR cryoablation OR ablation); limits: last 10 years, randomized controlled trials. RCTs, randomized controlled trials.

Fig. 1 summarises the PubMed search that selected the trials included in our analysis through the PRISMA algorithm. The trial by Baraki *et al*. [16] was excluded because only patients with endocarditis were enrolled.

Tables 1,2 (Ref. [3, 4, 5, 6, 7, 8]) summarise the comparative information about the 6 trials included in our analysis. In the 193 patients of the repair group, the mean age, weighted across the 6 trials, was 54.1 years, while the same figure in the 329 patients of the replacement group was 61.3 years.

**Table 1.** **Main characteristics of 6 controlled trials. The endpoint was death from any cause**.

First author and year of publication	Country	Patients’ characteristics			Follow-up length (months)	N° of patients in treatment ${\mathrm{arms}}^{†}$	
		All patients	Repair group	Replacement group		Repair group	Replacement group
Ejiofor, 2017 [3]	USA	Consecutive adult patients undergoing isolated tricuspid valve surgery	Age, 55.2 yrs	Age, 54.4 yrs	60 mos	2/18	4/39
			NYHA III–IV: 33%	NYHA III–IV: 49%			
Farag, 2017 [4]	Germany	consecutive patients undergoing surgical correction of tricuspid valve pathology	Age, 50.7 yrs	Age, 55.7 yrs	96 mos	6/41	8/68
			NYHA III–IV: NR	NYHA III–IV: NR			
Moutakiallah, 2018 [5]	Morocco	Consecutive rheumatic patients who underwent isolated tricuspid valve surgery	Age, 48.2 yrs		150 mos	4/15	2/11
			NYHA III–IV: 81%				
Oh, 2014 [6]	New Zealand	Consecutive adult patients undergoing isolated tricuspid valve surgery	Age, 48.9 yrs	Age, 46.9 yrs	25 mos	18/38	23/34
			NYHA III–IV: 33%	NYHA III–IV: 61%			
Patlolla, 2021 [7]	USA	Consecutive adult patients undergoing isolated tricuspid valve surgery	Age, 61.7 yrs	Age, 69.3 yrs	108 mos	34/60	88/163
			NYHA III–IV: 85%	NYHA III–IV: 69%			
Raikhelkar, 2013 [8]	USA	Consecutive adult patients undergoing isolated tricuspid valve surgery	Age, 51.8 yrs	Age, 60.2 yrs	120 mos	${\mathrm{7/21}}^{§}$	${\mathrm{3/14}}^{§}$
			NYHA III–IV: 41%	NYHA III–IV: 41%			

^†^These 12 arms include a total of 522 patients. ^§^The Kaplan-Meier curve was available for only 21 patients of the repair group (total number of patients, 27) and 14 patients of the replacement group (total number of patients, 28). Abbreviations: NYHA, New York Heart Association; NR, not reported; N°, number; yrs, years.

**Table 2.** **Devices employed in the 6 comparative trials**.

First author and year of publication	Devices used in the two patient groups	
	Repair group	Replacement group
Ejiofor, 2017 [3]	n = 18	n = 39
	-ring annuloplasty, 61%	-mechanical valve, 14%
	-bicuspidization, 17%	-bioprosthetic valve, 86%
	-De Vega annuloplasty, 17%	
	-vegetectomy, 5%	
Farag, 2017 [4]	n = 41	n = 68
	-standard ring annuloplasties, 26%	-mechanical or bioprosthetic valve, ${\mathrm{NR}}^{§}$
	-ring reconstructions or tightening using the De Vega technique, 14%	
	-valvuloplasty with pericardial patching or bicuscpidalization, 48%	
	-commissurotomy, 5%	
Moutakiallah, 2018 [5]	n = 15	n = 11
	-Carpentier Edwards ring, n = 30.7%	-mechanical valve (e.g., Sorin Bicarbon, ATS, Carbomdics), 55%
	-Carpentier Edwards ring, n = 32.73%	-bioprosthetic valve (e.g., Sorin Pericarbon More, Medtronic Hancock Tissue Valve II, SIM Epic Biocor), 45%
	-Carpentier Edwards ring, n = 34.20%	
Oh, 2014 [6]	n = 38	n = 34
	-De Vega technique, 29%	-mechanical valve, 79%
	-annuloplasty band, 55%	-bioprosthetic valve, 21%
	-non-annuloplasty valve repair, 16%	
Patlolla, 2021 [7]	n = 60	n = 163
	-prosthetic annuloplasty ring/band, 82%	-mechanical valve, 7%
	-suture annuloplasty, 18%	-bioprosthetic valve, 93%
Raikhelkar, 2013 [8]	n = 27	n = 29
	${\mathrm{NR}}^{§\,§}$	-mechanical valve, 3%
		-bioprosthetic valve, 97%

^§^Mechanical valves were used in patients aged <70 years, biological valves in patients aged ≥70 years. ^§§^The type of prosthesis used was based on the surgeon’s discretion and preference. Abbreviations: NYHA, New York Heart Association; NR, not reported.

### 3.2 Survival Analysis

**Fig. 2.**
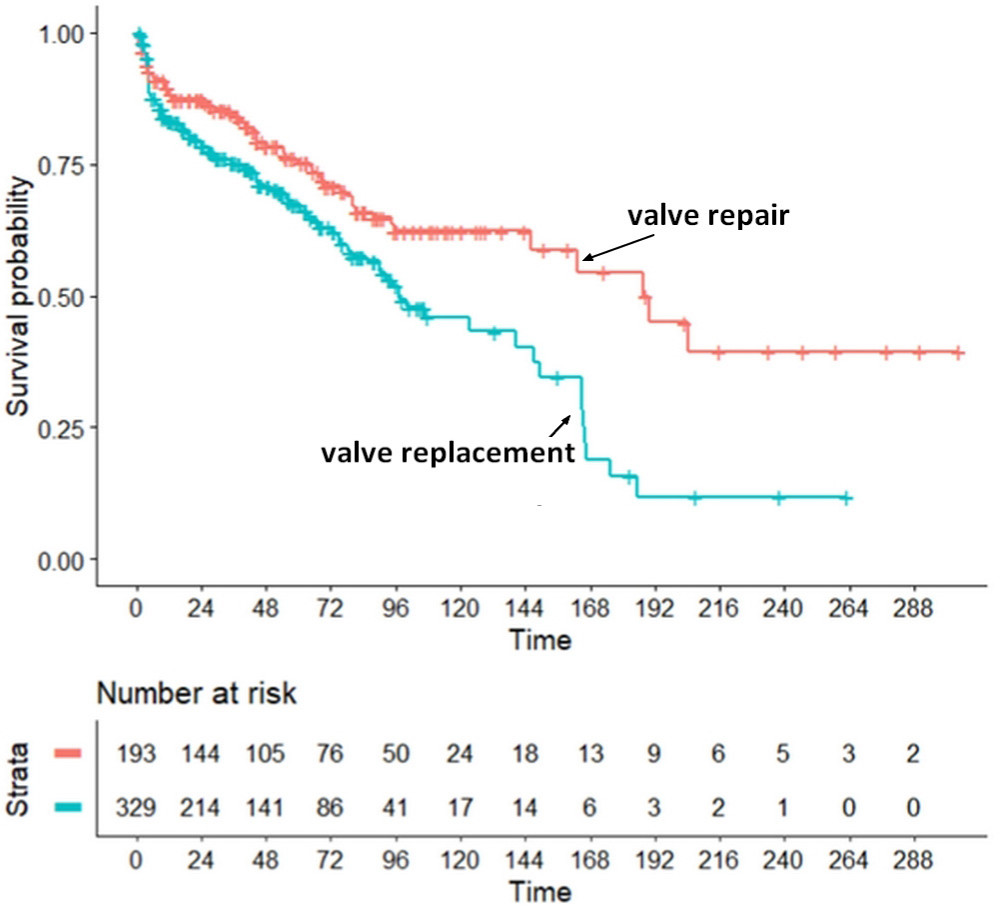
**Application of the IPDfromKM method: results of the primary analysis.** In red, patients from the 6 trials undergoing tricuspid valve repair (n = 193); In blue: patients from the 6 trials undergoing tricuspid valve replacement (n = 329). Time in months.

Our main survival analysis generated the results presented in Fig. 2. In this analysis, the survival outcomes determined in patients undergoing VREPA were found to be significantly better than those observed in patients undergoing VREPLA. The HR was 0.6098 (95%CI, 0.445 to 0.835; *p* = 0.002). As shown in Fig. 2, the mortality risk determined as a function of time showed a quite constant pattern over the first 4 years; then, the presence of a higher mortality in the replacement group became more and more pronounced.

### 3.3 Heterogeneity Assessment

**Fig. 3.**
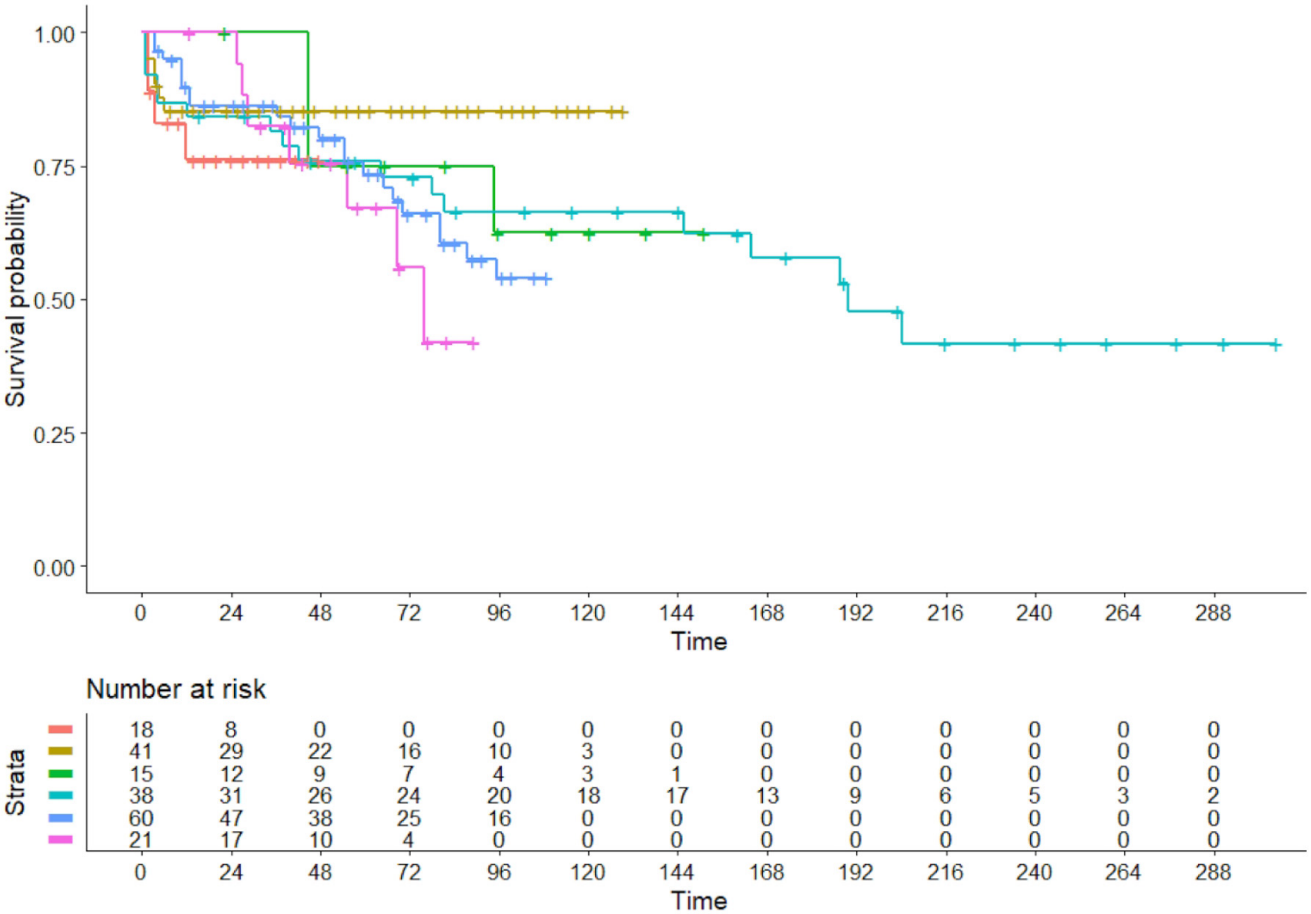
**Analysis of the 6 included trials: assessment of heterogeneity in the patient arms undergoing valve repair. **Time, in months.

In the case of VREPA, firstly the 6 curves (one for each trial) reporting the survival probability versus time were all plotted in a single graph (Fig. 3).

This graph provided a visual representation of the magnitude of between-trial differences and also showed how the trial-specific mortality risks varied over time. Concordance was 0.563 (standard error = 0.043); likelihood ratio test, 5.36 on 5 df (degrees of freedom) with *p* = 0.40; Wald test was 4.99 on 5 df, with *p* = 0.4. These indexes indicate that between-trial heterogeneity remained far from statistically significant.

**Fig. 4.**
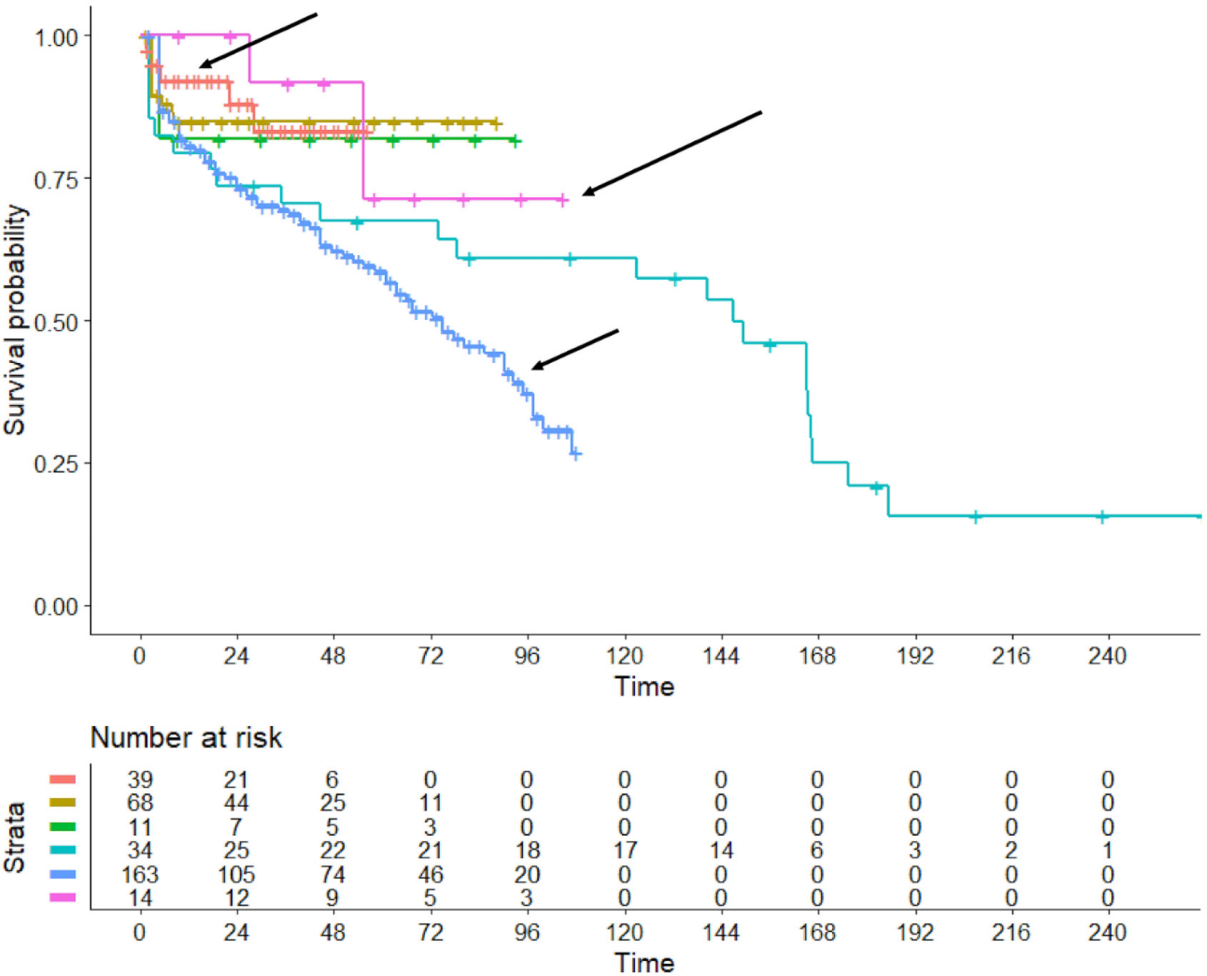
**Analysis of the 6 included trials: assessment of heterogeneity in the patient arms undergoing valve replacement**. The three black arrows denote the Kaplan-Meier curves for the studies by Ejiofor *et al*. [3]-in orange-, Patlolla *et al*. [7] -in blue-, and Raikhelkar *et al*. [8] -in purple-, in which bioprosthesic valves were used in at least 80% of patients. In Raikhelkar’s study [8], the long-term Kaplan-Meier curve was reported for only 14 patients out of the total number of 29 patients treated with valve replacement. Besides the above mentioned three cohorts (corresponding to the trials identified with References 3, 7, and 8, including a total of 39, 14, and 163 patients, respectively), the remaining three cohorts can be identified according to the Strata reported below the Kaplan-Meier graphs: the trials indicated by the remaining three colors (in which the total number of patients are 68, 11, and 34) correspond to References 4, 5, and 6, respectively. Time, in months.

Also in the case of VREPLA, the heterogeneity was studied through this approach (Fig. 4, Ref. [3, 7, 8]).

In this second case, the degree of heterogeneity was highly significant, reflecting the presence of substantial differences in outcomes across the 6 trials. Concordance was 0.587 (standard error = 0.026). The likelihood ratio test was 21.14 on 5 df, with *p* = 0.0008; Wald test = 18.59 on 5 df, with *p* = 0.002. A possible explanation for these findings of high heterogeneity between trials is that the two trials by Moutakiallah *et al*. [5] and Patlolla *et al*. [7] included patients with more advanced disease; indeed, the percentage of patients with preoperative New York Heart Association (NYHA) class III or IV was higher in these two trials than in the other four. Differences in patient age may also have contributed to this heterogeneity.

### 3.4 Limitations

Given the small sample size of the population of patients enrolled in the 6 clinical studies, our statistical analyses were limited to three key assessments: (i) a comparison of long-term survival between valve repair and valve replacement (Fig. 2); (ii) an assessment of heterogeneity among patients undergoing valve repair (Fig. 3); and (iii) an assessment of heterogeneity among patients undergoing valve replacement (Fig. 4). Although our results indicate a significantly better survival pattern for valve repair compared to valve replacement (Fig. 2), it is crucial to interpret this finding as a mainly descriptive result. In fact, our results are strongly influenced by the lack of a randomised design in these studies and by the unmatched baseline characteristics between the two groups of patients (as patients were not selected). It is important to note that randomised trials in this area are not currently available and are unlikely to become available in the near future. In addition, advanced matching techniques such as propensity score matching cannot be systematically applied due to the insufficient number of patients enrolled in these trials.

Certainly, the age of the patient at the time of the procedure is likely to be a critical factor influencing survival outcomes. For example, in inoperable patients, advanced age may indicate a longer duration of disease, making early repair at a younger age more beneficial. Conversely, interventions at a more advanced age may be less effective. It is important to note that our analysis has a limitation in that, due to the experimental design, we were not able to investigate the effect of these covariates. Finally, given the nature of our study, it is important to consider our findings regarding the age of the patients (mean age in the repair group, 54.1 years, versus 61.3 years in the replacement group) in order to better interpret our survival results.

Both analyses focused on heterogeneity (Figs. 3,4), the proportion of patients with a NYHA class of III or IV showed remarkable differences across the 6 trials (Table 2) and, as expected, this factor had a negative impact on prognosis. The percentage of patients who had a NYHA level III or IV was an important source of between-trial differences and likely explains the heterogeneity that we found especially across patients undergoing valve replacement.

## 4. Discussion

Some papers [17, 18, 19, 20] published between 2022 and 2023 provide important insights to place the results of our analysis in a broader context. As pointed out by Carino *et al*. [17], although several clinical studies have reported better outcomes of tricuspid valve repair with ring annuloplasty compared to suture techniques, their follow-up was usually limited to 10 years. In contrast, the study by Carino *et al*. [17] extended the follow-up to more than 15 years, confirming the superior results of tricuspid valve repair (based on ring implantation compared with suture techniques). According to this analysis, ring annuloplasty can therefore be considered the first option for tricuspid valve repair because of its well-documented superior durability. On the other hand, Piperata* et al*. [18] studied the long-term results of concomitant suture bicuspidation to treat mild or moderate tricuspid regurgitation at the time of mitral valve surgery. In this study, patients who underwent mitral valve surgery with concomitant tricuspid valve repair had similar 30-day and long-term survival, similar rates of permanent pacemaker implantation, and less progression of tricuspid valve regurgitation compared with patients who underwent mitral valve surgery alone. Although this is an interesting finding, it should be emphasized that in our analysis, isolated tricuspid surgery was a mandatory selection criterion for the 6 included trials.

Finally, the two studies published by Russo *et al*. [19, 20], despite their single-arm observational design, reported some interesting real-world experiences in isolated tricuspid interventions, which are helpful to better interpret our results. In the first retrospective observational study [19], the aim was to compare valve repair with valve replacement strategies in a total of 426 patients enrolled from 13 international centers. The results, based on 175 patient pairs matched by propensity score analysis, showed that isolated valve repair improved both early and late mortality with no difference in reoperation rates compared to replacement. The second study [20] was a multi-center retrospective study based on 13 international centers that included 406 adult patients who underwent isolated tricuspid valve procedures. The objective in this case was to compare isolated tricuspid valve surgery performed with a beating heart strategy with that performed with the standard arrested heart technique. The results showed that the beating heart approach was safe and resulted in a trend toward increased long-term survival and freedom from reoperation compared with the arrested heart approach. In summary, the results of these 4 clinical trials are particularly helpful in interpreting the results of our analysis (particularly those presented in Table 2). At the same time, since the specific type of surgical repair has been shown to influence clinical outcomes, the variability between the included studies has likely influenced our overall results and may be a contributing factor to the high heterogeneity found in some of our results.

In summary, the information from the recent literature that we have summarized above indicates that in patients with tricuspid regurgitation, valve repair is currently recognized as the preferred technique, but in some patients, this approach may not be feasible because of technical difficulties or because the patient’s disease has progressed to a stage where it is not recommended. While the clinical material selected for our analysis obviously reflects this controversy, the overall message from our main analysis (e.g., see Fig. 2) is clear, suggesting a better outcome with repair compared to replacement. However, it should be kept in mind that the 6 studies included in our analysis were not based on a randomized design, so these differences in outcome, although important, were likely influenced by the different characteristics of these patients at baseline. On the other hand, if we look at the published evidence, the literature published to date has aimed to synthesize the results reported in published studies rather than to identify a specific factor influencing these results. In this context, the meta-analysis by Chick *et al*. [1] is the most comprehensive study of its kind, as their analysis, based on 27 clinical trials, represents a systematic review of all trials evaluating tricuspid valve repair versus replacement. In contrast, our analysis was specifically focused on long-term outcomes and indeed concentrated on studies with at least 24 months of follow-up; as a result, we included only 6 clinical trials. It is worth noting that these 6 trials generally had a limited number of patients enrolled, which led us to interpret our findings primarily narratively.

The role of the IPDfromKM method in performing this type of analysis deserves some comment. Compared to a standard meta-analysis (e.g., a traditional binary meta-analysis or a network meta-analysis), the IPDfromKM method has several advantages. The most important is of a theoretical nature, because this method can handle censoring, whereas other types of meta-analyses of aggregate data are unable to make adjustments based on censoring. Thus, a common approximation in current “traditional” meta-analyses is to ignore both the length of follow-up and the effect of censoring, and to work with crude rates and 2 × 2 contingency tables to compare different treatments (i.e., numerator with the number of patients experiencing the endpoint over denominator with the total number of patients enrolled). The IPDfromKM method has an important advantage in overcoming this disadvantage, as it takes into account the presence of censored cases and also deals with the presence of different lengths of follow-up in different trials. The graphical presentation is also advantageous because it allows the time course of the endpoint to be examined visually. Another strength is the graphical approach to examining heterogeneity across pooled trials, which again uses a visual approach. The main limitation is that only a few people are familiar with the IPDfromKM method and, in general, with the reconstruction of individual patient data from Kaplan-Meier curves. In our view, these experiences, where the same datasets are examined in duplicate by a standard meta-analysis and by a re-analysis based on the IPDfromKM method (see also reference [14]), are worthwhile also because they increase the number of people familiar with the IPDfromKM method. In this context, the purpose of the present paper is also to encourage further debate about the pros and cons of the IPDfromKM method, beyond the considerations that have already been discussed in recent years, especially by oncologists.

## 5. Conclusions

Our analysis confirmed the methodological advantages of the IPDfromKM method in the indirect comparative analysis of multiple trials. These advantages include appropriate analysis of censored patients, original assessment of heterogeneity, and graphical presentation of the results, wherein individual patients retain an important role.

## Data Availability

The data sets generated and/or analyzed during the current study are not publicly available but are available from the corresponding author on reasonable request.

## References

[1] Chick W, Alkhalil M, Egred M, Gorog DA, Edwards R, Das R (2023). A Systematic Review and Meta-Analysis of the Clinical Outcomes of Isolated Tricuspid Valve Surgery. *The American Journal of Cardiology*.

[2] Zhu QM, Berry N (2023). Tricuspid Regurgitation: Disease State and Advances in Percutaneous Therapy. *European Cardiology*.

[3] Ejiofor JI, Neely RC, Yammine M, McGurk S, Kaneko T, Leacche M (2017). Surgical outcomes of isolated tricuspid valve procedures: repair versus replacement. *Annals of Cardiothoracic Surgery*.

[4] Farag M, Arif R, Sabashnikov A, Zeriouh M, Popov AF, Ruhparwar A (2017). Repair or Replacement for Isolated Tricuspid Valve Pathology? Insights from a Surgical Analysis on Long-Term Survival. *Medical Science Monitor: International Medical Journal of Experimental and Clinical Research*.

[5] Moutakiallah Y, Aithoussa M, Atmani N, Seghrouchni A, Moujahid A, Hatim A (2018). Reoperation for isolated rheumatic tricuspid regurgitation. *Journal of Cardiothoracic Surgery*.

[6] Oh TH, Wang TK, Sidhu K, Haydock DA (2014). Isolated tricuspid valve surgery at a single centre: the 47-year Auckland experience, 1965-2011. *Interactive Cardiovascular and Thoracic Surgery*.

[7] Patlolla SH, Schaff HV, Greason KL, Pochettino A, Daly RC, Frye RL (2021). Early Right Ventricular Reverse Remodeling Predicts Survival After Isolated Tricuspid Valve Surgery. *The Annals of Thoracic Surgery*.

[8] Raikhelkar J, Lin HM, Neckman D, Afonso A, Scurlock C (2013). Isolated tricuspid valve surgery: predictors of adverse outcome and survival. *Heart, Lung & Circulation*.

[9] Liu N, Zhou Y, Lee JJ (2021). IPDfromKM: reconstruct individual patient data from published Kaplan-Meier survival curves. *BMC Medical Research Methodology*.

[10] Messori A, Damuzzo V, Rivano M, Cancanelli L, Di Spazio L, Ossato A (2023). Application of the IPDfromKM-Shiny Method to Compare the Efficacy of Novel Treatments Aimed at the Same Disease Condition: A Report of 14 Analyses. *Cancers*.

[11] Messori A (2023). Reconstruction of individual-patient data from the analysis of Kaplan-Meier curves: the use of this method has extended from oncology to cardiology – List of 23 studies in cardiology- (preprint). Open Science Framework. https://osf.io/qejus.

[12] Page MJ, McKenzie JE, Bossuyt PM, Boutron I, Hoffmann TC, Mulrow CD (2021). The PRISMA 2020 statement: an updated guideline for reporting systematic reviews. *BMJ (Clinical Research Ed.)*.

[13] Messori A (2021). Synthetizing Published Evidence on Survival by Reconstruction of Patient-Level Data and Generation of a Multi-Trial Kaplan-Meier Curve. *Cureus*.

[14] Messori A, Fadda V, Romeo MR, Veneziano S, Trippoli S (2023). A Comparison of Statistical Analysis Between “Real” Patients Reported in Kaplan-Meier Curves and “Reconstructed” Patients Estimated Through the IPDfromKM Method: Analysis of Eight Trials Evaluating Catheter Ablation of Ventricular Tachycardia. *Cureus*.

[15] Core Team (2022). R: A language and environment for statistical computing. R Foundation for Statistical Computing, Vienna, Austria. https://www.R-project.org/.

[16] Baraki H, Saito S, Al Ahmad A, Fleischer B, Schmitto J, Haverich A (2013). Surgical treatment for isolated tricuspid valve endocarditis- long-term follow-up at a single institution. *Circulation Journal: Official Journal of the Japanese Circulation Society*.

[17] Carino D, Zancanaro E, Sala A, Ruggeri S, Lapenna E, Forno BD (2022). Durability of suture versus ring tricuspid annuloplasty: Looking at very long term (18 years). *Asian Cardiovascular & Thoracic Annals*.

[18] Piperata A, Van Den Eynde J, Pernot M, Busuttil O, Avesani M, Bottio T (2023). Long-term outcomes of concomitant suture bicuspidization technique to treat mild or moderate tricuspid regurgitation in patients undergoing mitral valve surgery. *European Journal of Cardio-thoracic Surgery: Official Journal of the European Association for Cardio-thoracic Surgery*.

[19] Russo M, Di Mauro M, Saitto G, Lio A, Berretta P, Taramasso M (2022). Outcome of patients undergoing isolated tricuspid repair or replacement surgery. *European Journal of Cardio-thoracic Surgery: Official Journal of the European Association for Cardio-thoracic Surgery*.

[20] Russo M, Di Mauro M, Saitto G, Lio A, Berretta P, Taramasso M (2022). Beating Versus Arrested Heart Isolated Tricuspid Valve Surgery: Long-term Outcomes. *The Annals of Thoracic Surgery*.

